# Inhibitory effect of carvacrol against *Alternaria alternata* causing goji fruit rot by disrupting the integrity and composition of cell wall

**DOI:** 10.3389/fmicb.2023.1139749

**Published:** 2023-02-20

**Authors:** Lunaike Zhao, Junjie Wang, Huaiyu Zhang, Peng Wang, Cong Wang, Yueli Zhou, Huanhuan Li, Shukun Yu, Rina Wu

**Affiliations:** Key Laboratory of Storage and Processing of Plant Agro-Products, School of Biological Science and Engineering, North Minzu University, Yinchuan, China

**Keywords:** carvacrol, *Alternaria alternata*, cell wall, antifungal mechanism, transcriptome

## Abstract

Goji (*Lycium barbarum* L.) is a widely planted crop in China that is easily infected by the pathogenic fungus *Alternaria alternata*, which causes rot after harvest. Previous studies showed that carvacrol (CVR) significantly inhibited the mycelial growth of *A. alternata in vitro* and reduced Alternaria rot in goji fruits *in vivo*. The present study aimed to explore the antifungal mechanism of CVR against *A. alternata*. Optical microscopy and calcofluor white (CFW) fluorescence observations showed that CVR affected the cell wall of *A. alternata*. CVR treatment affected the integrity of the cell wall and the content of substances in the cell wall as measured by alkaline phosphatase (AKP) activity, Fourier transform-infrared spectroscopy (FT-IR), and X-ray photoelectron spectroscopy (XPS). Chitin and β-1,3-glucan contents in cells decreased after CVR treatment, and the activities of β-glucan synthase and chitin synthase decreased. Transcriptome analysis revealed that CVR treatment affected cell wall-related genes in *A. alternata*, thereby affecting cell wall growth. Cell wall resistance also decreased with CVR treatment. Collectively, these results suggest that CVR may exert antifungal activity by interfering with cell wall construction, leading to impairment of cell wall permeability and integrity.

## 1. Introduction

Goji (*Lycium barbarum* L.) berry is one of the most widely cultivated and economically important fruit crops in northwest China (Wang et al., 2021). It is also a traditional Chinese herbal medicine, possessing vital biological activities such as anti-oxidation, anti-aging, and cancer prevention activities (Wang et al., 2010; Ma et al., 2022). However, goji berries are easily infected by *Alternaria alternata*, a pathogenic fungus that is responsible for black mold rot (Wang et al., 2018; Zhao et al., 2021; Supplementary Figure S1). The infected fruits not only cause important economic losses through postharvest decay, but also lead to health problems through contamination with various toxins produced by the pathogen (Wei et al., 2017; Xing et al., 2020). Fungicidal chemicals such as iprodione, tebuconazole, and mancozeb can effectively control postharvest rot caused by *A. alternata* (Yuan et al., 2019). However, the persistent application of synthetic fungicides has led to the emergence of drug resistance, environmental contamination, and health hazards for consumers caused by chemical residues (Fu et al., 2017; Zhou et al., 2018). Therefore, it is necessary to explore new alternatives to reduce the use of these traditional synthetic fungicides.

In recent years, single or blended compounds from plant essential oils have been widely considered to be a promising alternative to chemical fungicides owing to their potential antimicrobial activities and natural and environmentally friendly properties. Carvacrol (2-methyl-5-(1-methylethyl)-phenol, CVR), a significant component of oregano essential oil (Suntres et al., 2015), has been approved as a Generally Recognized As Safe (GRAS) food additive by the Food and Drug Administration (FDA) and the European Commission, and can therefore be used in a variety of foods and beverages (Ultee et al., 1999; De Vincenzi et al., 2022). Many studies have shown that CVR exhibits significant antifungal effects against a range of postharvest decay pathogens, such as *Pilidiella granati* (Thomidis and Filotheou, 2016), *Botrytis cinerea* (Zhang et al., 2019), *Fusarium verticillioides,* and *Aspergillus westerdijkiae* (Schloesser and Prange, 2019), *Penicillium verrucosum* (Ochoa-Velasco et al., 2017), and *Geotrichum citri-aurantii* (Serna-Escolano et al., 2019). Our previous study suggested that CVR can effectively suppress black mold rot caused by *A. alternata* and maintain the postharvest quality of goji berries (Wang et al., 2019). Meanwhile, Abbaszadeh et al. (2014) demonstrated that CVR can inhibit the growth of *A. alternata in vitro*. However, the mechanisms of CVR against *A. alternata* have yet to be elucidated.

The antifungal mechanism of CVR has been extensively studied. CVR treatment can disrupt the cell morphology of *Botrytis cinerea* and alter the permeability of the cell membrane (De Souza et al., 2015). Kim et al. (2019) found that reactive oxygen species (ROS) generated by CVR damaged the cell membrane and even caused cell death of *Raffaelea quercus-mongolicae* and *Rhizoctonia solani*. Furthermore, Li J. et al. (2021) reported that CVR treatment could result in the death of *Botryosphaeria dothidea*-like cells by significantly inhibiting mitochondrial activity and respiration rates. However, to date, no studies have shown whether CVR affects the cell wall of *A. alternata*, and its mechanism of action against this pathogenic fungus has not been reported. Therefore, the impact of CVR on the cell wall of *A. alternata* requires further examination.

Accordingly, the objective of the present research was to investigate the effect of CVR treatment on the morphology and integrity of the cell wall of *A. alternata in vitro*. Meanwhile, the functional groups and surface chemical composition of the mycelia of the cell wall were measured by FT-IR and XPS, respectively. Furthermore, the major polysaccharide content of chitin and β-1,3-glucan and the enzyme’s activity related to these polycarbohydrates metabolism were determined. Further study was conducted to evaluate the effect of the CVR treatment on transcriptome and gene expressions using RNA-seq and qRT-PCR techniques. In addition, the growth of *A. alternata* in different environments was studied to explore the stress resistance response. The study verified the inhibition mechanism of CVR on the cell wall of *A. alternata*, and laid the groundwork for the application of CVR to control other fruit fungal diseases.

## 2. Materials and methods

### 2.1. Fungal strain

According to the method of Jiang et al. (2019), a strain isolated from goji berries showing black mold rot symptoms was identified as *A. alternata* causing goji postharvest rot by analyzing morphological characteristics (Supplementary Figure S1) and nucleotide sequences containing the internal transcribed spacer rDNA region (*ITS*) (Supplementary Figure S2), β-tubulin (*tub2*) (Supplementary Figure S3), endopolygalacturonase (*endoPG*) (Supplementary Figure S4), and Alternaria major allergen (*Alta1*) (Supplementary Figure S5). The gene sequences of the isolated pathogen were submitted to GenBank (accession nos. MN653245.1, MN702782.1, MN698284.1, and MN702781.1 for *ITS*, *tub2*, *endoPG*, and *Alta1*, respectively). The strain of *A. alternata* was cultured on potato dextrose agar (PDA) for 8 days at 28 ± 1°C (Zhao et al., 2021).

### 2.2. Preparation of CVR treatments

*Alternaria alternata* was treated with CVR (purity ≥98.0%, Tokyo Kasei Kogyo Co., Ltd., Japan) by using the fumigation method (Cardiet et al., 2012). Briefly, 6-mm inoculum disks of *A. alternata* were cut from the leading edge of the fungal culture on PDA plates using a hole punch, placed in the center of new plates, and incubated at 28°C and 90% relative humidity (RH) for 2 days. Next, a 60-mm filter paper sheet was placed on the lid of each plate and 1 ml dissolved CVR (containing 5% Tween-80) was added to the filter paper so that the concentration of volatile CVR in the sealed plates was 0, 0.06, 0.12, and 0.24 μl/ml, respectively. The edge of each plate was sealed with parafilm and the plates were incubated upside down at 28°C and 90%RH for 3 days. The colony was divided into inside and outside parts, as shown in Supplementary Figure S6, simulating the effect of CVR on the formed mycelia (inside) and on the newly grown mycelia (outside), respectively. The outside and inside mycelia were scraped and weighed to 1 g portions, then wrapped in tin foil, rapidly frozen in liquid nitrogen, and stored at −80°C for later use.

### 2.3. Effect of CVR on cell wall integrity of *A. alternata*

#### 2.3.1. Microscopy observations

Changes in mycelial morphology of *A. alternata* after CVR treatment were observed by optical microscopy. The effects of CVR on the integrity of the cell wall of *A. alternata* were analyzed by calcofluor white (CFW) staining coupled with fluorescence microscopy (Ouyang et al., 2019). The original mycelia and newly formed mycelia treated with different concentrations of CVR were picked and stained with 10 μl of 10% KOH and 10 μl CFW.

#### 2.3.2. Extracellular alkaline phosphatase activity assay

The extracellular AKP activity of *A. alternata* mycelia treated with different concentrations of CVR was assayed using an AKP kit (Beijing Solarbio Science and Technology Co., Ltd., China) according to the manufacturer’s instructions. Enzyme activity was expressed as U/mg protein.

#### 2.3.3. Fourier transform-infrared spectroscopy

The FT-IR of the *A. alternata* mycelia treated with different concentrations of CVR was determined according to the method of Li Q. et al. (2021). Briefly, the mycelia were lyophilized and ground to a powder. The samples and KBr were mixed at 1:100 (M:M) and placed in an agate mortar. After fine grinding and drying, the mixture was pressed into thin slices and the surface functional groups were determined by FT-IR spectrophotometer. The FT-IR wavelength range was 400–4,000 cm^−1^ and the background was determined using pure KBr.

#### 2.3.4. X-ray photoelectron spectroscopy

The relative substance content of *A. alternata* mycelia treated with different concentrations of CVR was determined according to method of Dague et al. (2008). Samples were lyophilized and ground into a powder. The powder was pressed into thin slices and etched with argon gas for 30 s. The test parameters were set as follows: energy, 1486.8 eV; test spot area, 400 μm; tube voltage, 15 kV; tube current, 10 mA; and background vacuum, 2 × 10^−9^ mbar. The binding energy range for the collection measurement scan was 1,100 eV to 0. The analyzer pass energy was 50 eV with a step of 1.00 eV.

### 2.4. Effect of CVR on polysaccharides in the cell wall of *A. alternata*

#### 2.4.1. Determination of β-1,3-glucan content

Referring to the method of Fortwendel et al. (2008), the content of β-1,3-glucan was determined by aniline blue fluorescence. A total of 50 mg of mycelia was harvested, washed with 0.1 mol/l NaOH, and lyophilized to powder. Next, 5 mg lyophilized mycelia powder was resuspended in 250 μl of 1 mol/l NaOH and reacted at 52°C for 30 min. Subsequently, 50 μl sample extract was transferred to a 96-well microtiter plate, and 210 μl aniline blue mixture was added to each well. After incubation at 52°C for 30 min, the plate was cooled at room temperature for 30 min to decolorize the unbound dye. The fluorescence of the bound dye complexes was detected with a microplate reader at an excitation wavelength of 450 nm and an emission wavelength of 460 nm. A β-1,3-glucan standard curve was created with a concentration range of 10 to 50 μg/ml and was used quantify the amount of β-1,3-glucan present in each sample.

#### 2.4.2. Measurement of β-1,3-glucan synthase activity

β-1,3-glucan synthase activity was measured referring to the method of Belewa et al. (2017). Microsomal proteins were obtained by crushing mycelia and pulverizing by ultracentrifugation. The microsomal proteins were resuspended in 500 μl buffer (1 mmol/l EDTA; 1 mmol/l DTT; 33% (v/v) glycerol; 50 mmol/l Tris–HCl, pH 7.5) and stored at −80°C until use. A 50 μl reaction system was established consisting of 50 mmol/l Tris–HCl (pH 7.5), 20 μmol/l GTP, 4 mmol/l EDTA, 0.5% Brij-35, 6.6% glycerol, 2 mmol/l UDP-Glc, and 100 μg microsomal protein. After incubation at 25°C for 30 min, the reaction was stopped by adding 10 μl of 6 mol/l NaOH. The dextran produced by the previous process was dissolved at 80°C for 30 min. The glucan content was measured by the aniline blue method. One unit of enzymatic activity (U/mg protein) is defined as 1 mg protein catalyzed to produce 1 μg of glucan.

#### 2.4.3. Determination of chitin content

Chitin content was determined by the method of Belewa et al. (2017). A total of 5 mg lyophilized mycelia was mixed with 3 ml of 1 mol/l KOH and incubated at 130°C for 1 h. Then, 8 ml of 70% ethanol was added to the mixture and the mixture was washed with 13.3% (w/v) cellulose acetate, 10 ml of 40% ethanol, and distilled water. The mixture was centrifuged at 1500 *g* for 5 min at 2°C. The resulting precipitate was added into a buffer containing 0.5 ml sterile double-distilled water mixed with 0.5 ml of 5% (w/v) sodium nitrite and potassium bisulfate, and then, the reaction system was centrifuged at 1500 *g* for 2 min. The 3-methyl-2-benzothiazolinone hydrochloride (MBTH) method was used to determine the glucosamine in the supernatant. The concentration of chitin in mycelia was determined by measuring the concentration of glucosamine produced by decomposition. Standard curves were constructed using various concentrations (5–25 μg/ml) of glucosamine.

#### 2.4.4. Measurement of chitin synthase

Chitin synthase activity was measured according to the method of Mellado et al. (2003). Microsomal proteins were obtained as described by the method in section 2.4.2. The microsomal proteins were resuspended in 400 μl Tris–HCl buffer (pH 7.5, 50 mmol/l, containing 30% (v/v) glycerol) and stored at −80°C until use. Subsequently, 100 μl of 2× buffer (32 mmol/l Tris–HCl, pH 7.5; 4.3 mmol/l magnesium acetate; 32 mmol/l GlcNAc; 1.1 mmol/l UDP-GlcNAc; and 4 μg trypsin) and 100 μg microsomal protein were added to a 96-well plate pretreated by 100 μl of a solution of 50 μg/ml WGA. The plates were incubated at 25°C for 90 min to synthesize chitin. Next, 20 μl of 50 mmol/l EDTA and 100 μl of 1 μg/ml WGA horseradish peroxidase conjugate were added to each well. The plate was shaken vigorously for 15 min, washed five times with double-distilled water, and then 100 μl TMB peroxidase reagent (1 ml 20% H_2_O_2_; 9 ml sterile distilled water; and 1 mg TMB dissolved in sterile water containing 20% H_2_O_2_) was added to the wells. The reaction was stopped by adding 100 μl of 1 mol/l sulfuric acid and the absorbance was measured at 450 nm. One unit of enzymatic activity (U/mg protein) is defined as 1 mg protein catalyzing the production of 1 μg chitin.

#### 2.4.5. Assays for chitinase and β-1,3-glucanase

Chitinase and β-1,3-glucanase activities of *A. alternata* mycelia were assayed according to the instructions of the chitinase kit and the β-1,3-glucanase kit (Suzhou Comin Biotechnology Co., Ltd., Jiangsu, China), respectively. Enzyme activity is expressed as U/mg protein.

### 2.5. Transcriptomic and quantitative real-time PCR analyses

*Alternaria alternata* were cultured on PDA for 3 days and then fumigated with or without 0.06 μl/ml CVR for 48 h. Referring to the method described in section 2.2, the external mycelia were rapidly frozen in liquid nitrogen and stored at −80°C for transcriptome analysis. The transcriptome information had been deposited at the GenBank Sequence Read Archive (SRA) database, with accession numbers SRR23083390, SRR23083389, SRR23083388, SRR23083387, SRR23083386, and SRR23083385. Total RNA was extracted with a TRIzol kit (Invitrogen, Carlsbad, CA, United States). cDNA library construction and Illumina RNA sequencing were performed by Microeco Technology Co., Ltd., Shenzhen, China (Parkhomchuk et al., 2009). Differentially expressed genes (DEGs) were screened by fold change (FC) >2 and *p*-value <0.05. Gene Ontology (GO) classification was conducted using clusterProfiler. Kyoto Encyclopedia of Genes and Genomes (KEGG) pathway annotation was performed by KOBAS 2.1 (Xie et al., 2011).

For qRT-PCR, total RNA was extracted by a fungal RNA kit (Omega Bio-Tek, Inc.) and reverse transcribed using the ABScript III rt. master mix kit (ABclonal Technology Co., Ltd). qRT-PCR was performed using the Genious 2× SYBR Green rapid qPCR mix kit (ABclonal Technology Co., Ltd). The β-tubulin gene was used as an internal control and relative gene expression was calculated using the 2^−∆∆CT^ method. Primer sequences are listed in Supplementary Table S1.

### 2.6. Analysis of the effects of CVR on stress resistance in *A. alternata*

For stress resistance assays, the inner and outer mycelia of *A. alternata* treated with CVR (0, 0.06, 0.12, and 0.24 μl/ml) were made into 6-mm inoculum disks, respectively, and applied to plates of PDA without or with each stress substance (Zhang et al., 2020): (i) 0.2 mg/ml Congo red or 0.005% SDS for cell wall-perturbing stress, (ii) 0.5 mol/l NaCl for osmotic stress, (iii) 0.01 mol/l H_2_O_2_ for oxidative stress, and (iv) 32°C for high-temperature stress. Plates were incubated at 28°C for 5 days, except for high-temperature stress plates, which were incubated at 32°C for 5 days. The colony diameter was measured vertically and inhibition rates were calculated; samples without treatment were used as controls.

### 2.7. Statistical analysis

All data were expressed as the mean ± standard deviation of three independent replicates. SPSS software version 16.0 was used for one-way analysis of variance (ANOVA) and a *p*-value <0.05 was deemed significant.

## 3. Results

### 3.1. Effect of CVR on cell wall integrity of *A. alternata*

**Figure 1:**
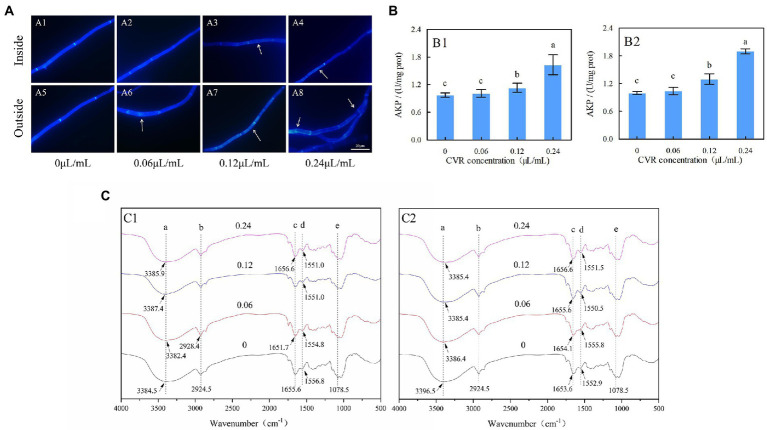
*Alternaria alternata* treated with CVR and observed under a fluorescence microscope after CWF staining (×100 magnification). **(A)** CK treatment of inside **(A1)** and outside **(A5)** parts of mycelia; 0.06 μl/ml CVR treatment of inside **(A2)** and outside **(A6)** parts of mycelia; 0.12 μl/ml CVR treatment of inside **(A3)** and outside **(A7)** parts of mycelia; 0.24 μl/ml CVR treatment of inside **(A4)** and outside **(A8)** parts of mycelia, respectively, **(B)** The effect of AKP enzyme activity after CVR treatment of inside **(B1)** and outside **(B2)** parts of mycelia, and **(C)** Influence of CVR on cell walls of *A. altern*ata. Different letters represent significant differences at the level of *p* < 0.05. FT-IR spectra of mycelia treated with CVR at 0, 0.06, 0.12, and 0.24 μl/ml of outside **(C1)** and inside **(C2)** parts of mycelia.

Microscopy observations showed that CVR treatment affected the morphology of internal and external mycelia of *A. alternata* (Supplementary Figure S7). The inner and outer mycelia without treatment were straight and thick. However, the mycelia treated with CVR gradually became thinner, the cell contents gradually clouded, the mycelia broke, and the cell wall collapsed (Supplementary Figures S7H,P). The integrity of the cell wall was detected by CFW staining using a fluorescence microscope. As shown in Figure 1A, the surface of *A. alternata* mycelia in the control sample had uniform fluorescence with bright cell spacing. In contrast, the fluorescence distribution on the surface of the mycelia treated with CVR was not uniform, and the brightness of the treated mycelia interval was decreased. With increasing concentrations of CVR, the degree of uneven fluorescence on the mycelia surface deepened. Furthermore, deformities occurred in the mycelia treated with 0.24 μl/ml CVR. These results indicated that CVR may cause damage and alterations to the integrity of the mycelial cell wall of *A. alternata*.

Determination of AKP activity in *A. alternata* treated with CVR can reflect the damage to the cell wall. The AKP activity of the treatment group was always higher than that of the control group (Figure 1B). After treatment with 0.06, 0.12, and 0.24 μl/ml CVR, the AKP activity of the inside mycelia increased by 3.69, 16.23, and 67.45%, respectively (Figure 1B1), while that of the outside part increased by 4.23, 30.21, and 90.26%, respectively (Figure 1B2).

### 3.2. FT-IR analysis of CVR-treated mycelia

To determine the structural differences in the mycelial cell wall between the control and CVR-treated groups, FT-IR was used to analyze the functional groups of these samples. Each site in the figure represented different hanging energy groups; 3396.5 cm^−1^ was O-H, 2924.5 cm^−1^ was C-H, 1653.6 cm^−1^ was amide I, 1552.9 cm^−1^ was amide II, and 1078.5 cm^−1^ was the C-C bond in glucose (Figure 1C). The bonds of amide I and amide II are characteristic protein bonds. After CVR treatment, external peak deflections were observed at sites a, c, and d in both inside and outside parts of the mycelia of *A. alternata*. This indicated that CVR caused changes in the OH functional group and proteins in the mycelia of *A. alternata*.

### 3.3. CVR treatment decreased the polysaccharide content of the cell wall surface

C, N, and O are the three main elements in the cell wall. XPS analyses were performed to determine the changes in these elements following CVR treatment. Typical XPS spectra between treated and control groups are presented in Supplementary Figure S8. The C_1s_ peak on the surface of *A. alternata* can be decomposed into C-(C,H) at 284.4 eV, C-(O,N) at 286.3 eV, and C=O bond at 288.0 eV. The O_1s_ peak had two components—one belonged to the O-C bond at 532.7 eV and the other belonged to the O=C bond at 531.4 eV. The N_1s_ peak was composed of two components: one component at 399.9 eV was attributed to amine or amide function, and the other component at 401.4 eV was attributed to protonated nitrogen. According to the formula, [N/C]_obs_ = 0.279(C_Pr_/C); [N/C]_obs_ = 0.279(C_Pr_/C); [C/C]_obs_ = (C_Pr_/C) + (C_Ps_/C) + (C_Lp_/C) = 1, the values of C_Pr_, C_Lp_, and C_Ps_ can be calculated (Table 1). C_Ps_ is the relative content of polysaccharide substance. The content of C_Ps_ in *A. alternata* was decreased after CVR treatment, indicating that the content of polysaccharide substances in the *A. alternata* cell wall decreased after CVR treatment.

**Table 1:** Surface chemical composition of mycelia of *A. alternata* treated with CVR at 0, 0.06, 0.12, and 0.24 μl/ml, measured by XPS.

Mycelia	CVR concentration (μg/mL)	%C	%N	%O	N/C	O/C	N/O	C_Pr_	C_Ps_	C_Lp_
Inside	0	69.08	2.95	27.97	0.04	0.40	0.11	10.57	29.45	29.05
	0.06	69.19	3.24	27.57	0.05	0.40	0.12	11.61	28.57	29.01
	0.12	69.85	3.31	27.84	0.05	0.40	0.12	11.86	28.79	28.19
	0.24	70.34	3.19	26.46	0.05	0.38	0.12	11.43	27.30	31.60
Outside	0	70.76	2.50	26.74	0.04	0.38	0.09	8.96	28.60	33.19
	0.06	70.31	2.97	26.72	0.04	0.38	0.11	10.65	27.92	31.74
	0.12	70.65	3.78	25.57	0.05	0.36	0.15	13.55	25.41	31.69
	0.24	70.37	4.16	25.47	0.06	0.36	0.16	14.91	24.76	30.70

### 3.4. Effects of CVR on β-1,3-glucan and chitin contents

**Figure 2:**
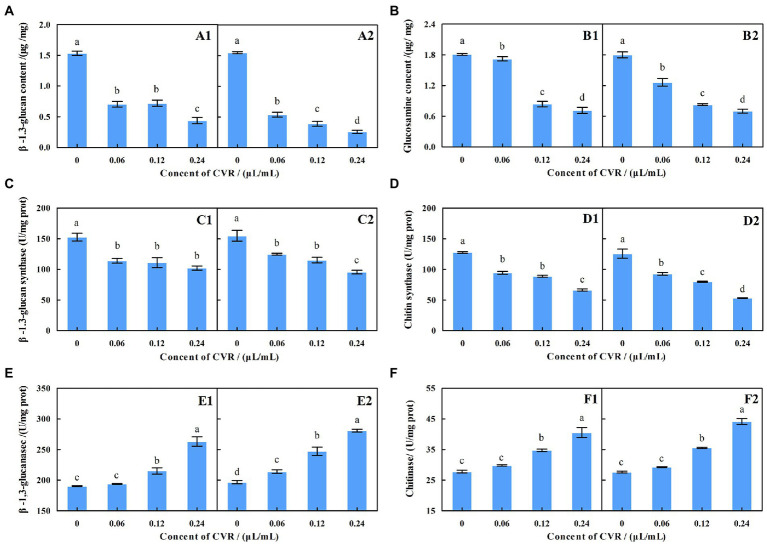
Effect of CVR on the content of β-1,3-glucan **(A)** in inside **(A1)** and outside **(A2)**, parts of mycelia, respectively; the content of chitin **(B)** in inside **(B1)** and outside **(B2)** parts of mycelia, respectively; the β-glucan synthase **(C)** activity in inside **(C1)** and outside **(C2)** parts of mycelia, respectively; the production of chitin synthase activity **(D)** in inside **(D1)** and outside **(D2)** parts of mycelia, respectively; the production of β-1,3-glucanase activity **(E)** in inside **(E1)** and outside **(E2)** parts of mycelia, respectively; and the production of chitinase activity **(F)** in inside **(F1)** and outside **(F2)** parts of mycelia, respectively. Different letters represent significant differences at the level of *p* < 0.05.

The contents of β-1,3-glucan and chitin in *A. alternata* were significantly reduced after CVR treatment (Figures 2A,B). After treatment with 0.06, 0.12, and 0.24 μl/ml CVR, the β-1,3-glucan content in the inner mycelia decreased by 54.01%, 53.03, and 71.43%, respectively, compared with the control, while that in the outer mycelia decreased by 65.43, 75.05, and 83.50%, respectively, compared with the control. The content of chitin in the inner mycelia decreased by 4.81, 53.66, and 60.49%, respectively, after treatment with 0.06, 0.12, and 0.24 μl/ml CVR, while chitin content in the outer mycelia decreased by 29.86, 54.05, and 61.08%, respectively. This indicated that CVR treatment can destroy the cell wall structure of *A. alternata* by reducing the contents of β-1,3-glucan and chitin.

### 3.5. Effects of CVR on β-glucan synthase and chitin synthase

The ability of *A. alternata* to synthesize β-1,3-glucan and chitin was reduced after CVR treatment (Figures 2C,D). After treatment with 0.06, 0.12, and 0.24 μl/ml CVR, the amount of β-glucan produced by the internal mycelia was 74.61, 72.86, and 66.91% of the control group, respectively, while that of the external mycelia was 80.59, 74.42, and 61.67% of the control group, respectively. The content of glucan produced by the inner mycelia was 73.93, 69.65, and 51.88% of the control group, respectively, and that of the outer mycelia was 73.86, 63.59, and 42.36% of the control group, respectively. This demonstrated that CVR can reduce the content of β-1,3-glucan and chitin by reducing the activity of β-glucan synthase and chitin synthase in the mycelia of *A. alternata.*

### 3.6. Effects of CVR on β-1,3-glucanase and chitinase

Figures 2E,F show the effect of CVR treatment on β-1,3-glucanase and chitinase activities in the mycelia of *A. alternata*. The higher the concentration of CVR, the larger the increased effect on the activities of β-1,3-glucanase and chitinase. In general, CVR treatment accelerated the degradation of chitin and β-1,3-glucan in the cell wall of *A. alternata*.

### 3.7. Transcriptome sequencing and analysis of DEGs

**Figure 3:**
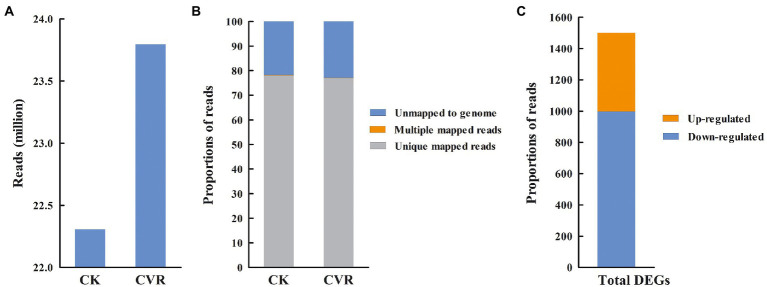
Summary information for transcriptome data. **(A)** High-quality clean reads, **(B)** The proportion of high-quality clean reads of mapped to unique genes, multiple genes, and unmapped genes, and **(C)** The number of differentially expressed genes (DEGs).

Sequencing data statistics showed Q20 > 97.25% and Q30 > 92.58%, which indicated that the sequencing quality was good. After eliminating primer and adapter sequences and filtering out poor-quality reads, 23.79 and 22.30 million clean reads were obtained in the CVR-treated and control samples, respectively (Figure 3A). Based on the clean reads of CVR-treated samples, approximately 76.91% were mapped to the *A. alternata* unique genes, 0.12% were mapped to multiple genes, and 22.97% did not map to known genes (defined as unmapped), respectively. Similar results were obtained in the control group samples (Figure 3B). The transcriptomes of *A. alternata* with CVR treatment and the control treatment were analyzed and a total of 1,492 DEGs were found (FC ≥2, *p* < 0.05), which included 504 upregulated DEGs and 988 downregulated DEGs (Figure 3C).

### 3.8. Go term enrichment and KEGG pathway analysis of DEGs

**Figure 4:**
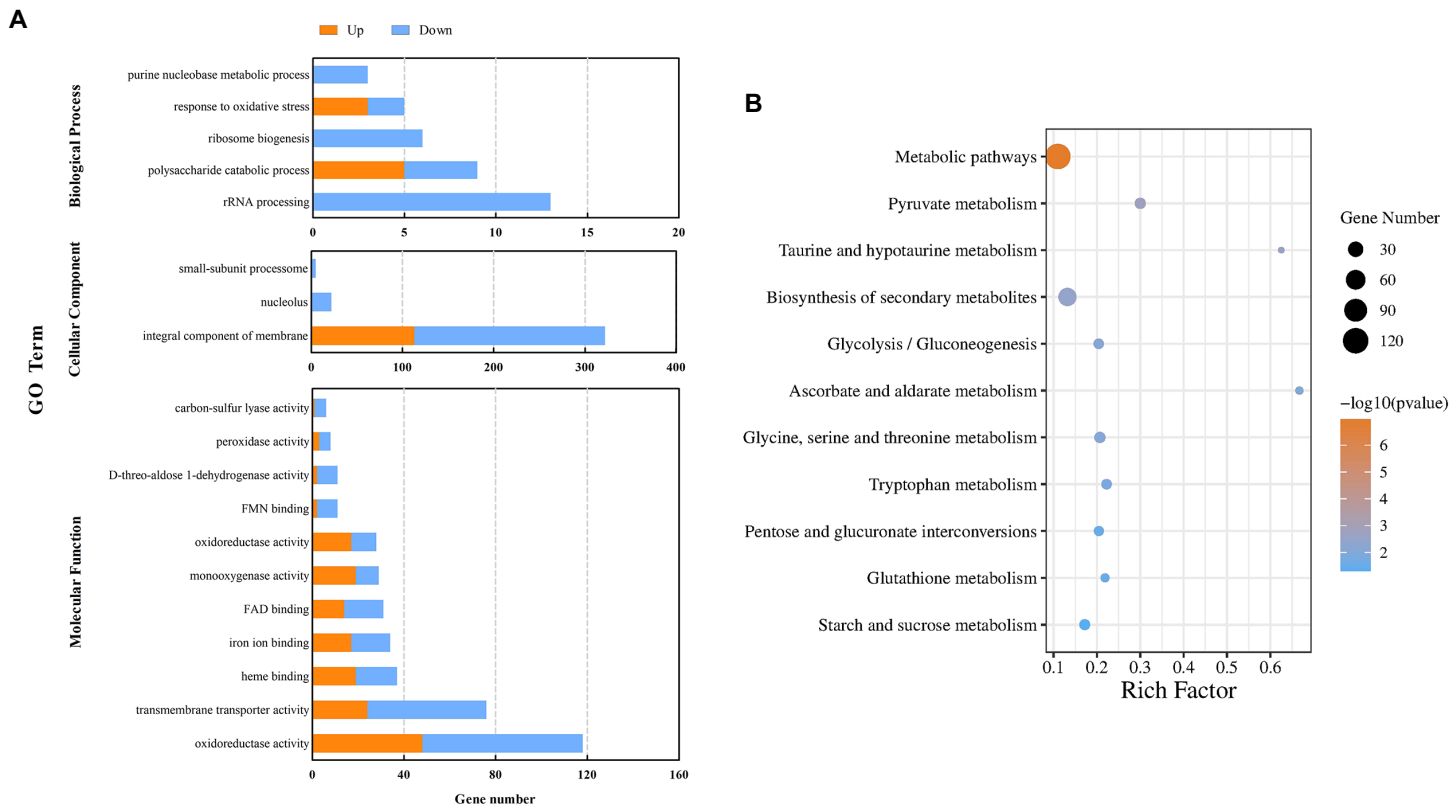
GO analysis of DEGs in the control sample and after treatment with CVR **(A)**. KEGG pathways and DEGs after treatment with CVR **(B)**.

GO enrichment analysis was performed on all DEGs (*p* < 0.05), and the DEGs were classified from three aspects: biological process, molecular function, and cellular component (Figure 4A). The DEGs in biological process were enriched in the terms of rRNA processing (13 DEGs), polysaccharide catabolic process (9), ribosome biogenesis (6), response to oxidative stress (5), and purine nucleobase metabolic process (3). The DEGs in molecular functions included oxidoreductase activity (118), transmembrane transporter activity (76), heme binding (37), iron ion binding (34), FAD binding (31), monooxygenase activity (29), oxidoreductase activity (28), FMN binding (11), D-threo-aldose 1-dehydrogenase activity (11), peroxidase activity (8), and carbon-sulfur lyase activity (6). It was also observed that DEGs classified in cellular component were enriched in the terms of integral component of membrane (322) and nucleolus (22).

The DEGs were further annotated against the KEGG database (Figure 4B) and were grouped into 84 KEGG pathways, including 5 in cell process, 2 in environmental information processing, 10 in genetic information processing, and 67 in metabolic pathways. Among them, 13 KEGG pathways were considered to be significantly enriched (*p* < 0.05). The significantly enriched KEGG pathways and the number of DEGs in each were metabolic pathways (120 DEGs), pyruvate metabolism (12), taurine and hypotaurine metabolism (5), biosynthesis of secondary metabolites (49), glycolysis/gluconeogenesis (10), ascorbate and aldarate metabolism (6), glycine, serine, and threonine metabolism (12), tryptophan metabolism (10), pentose and glucuronate interconversions (9), glutathione metabolism (7), and starch and sucrose metabolism (11). These results showed that CVR altered the overall metabolic process(es) of *A. alternata*.

### 3.9. Changes in gene expression of starch and sucrose metabolism signaling pathways

**Figure 5:**
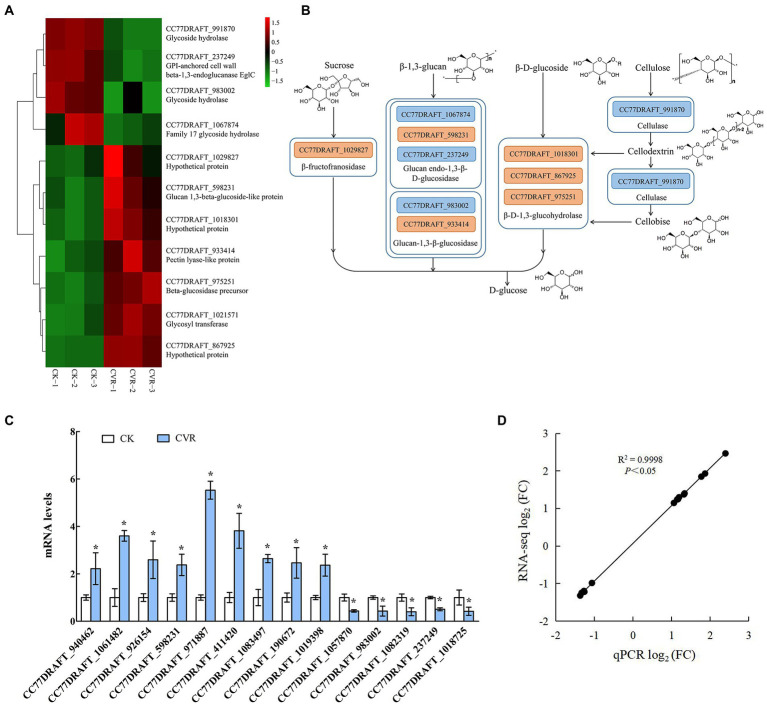
RNA-Seq influence of CVR treatment on metabolism of polysaccharide substances. **(A)** DEGs in sucrose metabolism signaling pathway, **(B)** Possible mechanism of CVR treatment regulating sucrose metabolism signaling pathway, **(C)** Relative expression levels of candidate genes by qRT-PCR. The data were analyzed by *t*-test; * represents significant difference (*p* < 0.05), and **(D)** Correlation between results of qRT-PCR and results of RNA-seq.

The starch and sucrose metabolism pathway is related to the changes of polysaccharides in the cell wall of *A. alternata* treated with CVR. There were 7 upregulated genes and 4 downregulated genes in the starch and sucrose metabolism pathway after CVR treatment (Figure 5A). Among these DEGs, the gene *CC77DRAFT_1029827* (hypothetical protein) regulating β-fructofuranosidase was markedly upregulated. Two downregulated genes—*CC77DRAFT_1067874* (family 17 glycoside hydrolase) and *CC77DRAFT_237249* (GPI-anchored cell wall beta-1,3-endoglucanase EglC)—and an upregulated gene, *CC77DRAFT_598231* (glucan 1,3-beta-glucoside-like protein), are involved in the regulation of glucan endo-1,3-β-D-glucosidase. Meanwhile, the upregulated gene *CC77DRAFT_933414* (Pectin lyase-like protein) and the downregulated gene *CC77DRAFT_983002* (glycoside hydrolase) jointly regulate glucan-1,3-β-glucosidase. The gene *CC77DRAFT_991870* (glycoside hydrolase) regulating cellulase was markedly downregulated and the gene *CC77DRAFT_1021571* (glycosyltransferase) regulating glycogen phosphorylase was clearly upregulated. All 10 genes except *CC77DRAFT_1021571* were related to the degradation of cell wall polysaccharides, which indicated that CVR treatment significantly affects the metabolism of polysaccharides in *A. alternata* (Figures 5A,B).

### 3.10. Changes in genes that may regulate the metabolism of cell wall growth and verification of the expression of candidate genes

Through the analysis of DEGs, 14 genes that may be related to cell wall integrity and substance content of *A. alternata* (Supplementary Table S1) were screened by qRT-PCR. The qRT-PCR data (Figure 5C) were consistent with the RNA-seq data, and the correlation coefficient R^2^ = 0.9998 (Figure 5D) verified the expression of the 14 candidate genes. These results indicated that the transcriptome data were reliable.

### 3.11. Effects of CVR on stress resistance

**Figure 6:**
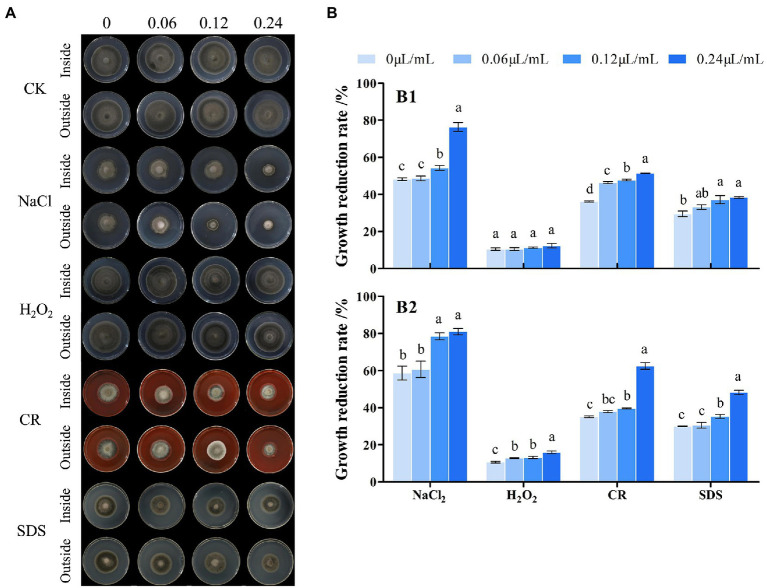
Influence of CVR treatment on stress resistance. Image of cultured under different conditions **(A)**. Growth reduction rate (%), **(B)** of inside **(B1)** and outside **(B2)** fungal colonies grown at 25°C for 8 days on PDA plates supplemented with NaCl (0.5 M), CR (Congo red 5 μg/ml), H_2_O_2_ (1.5 mM), and SDS (0.1 mg/ml), respectively. Different letters represent significant differences at the level of *p* < 0.05.

The inside and outside parts of the mycelia of *A. alternata* after treatment with CVR were applied to culture media containing different stress substances. The antifungal effect was observed to determine the resistance of the *A. alternata* cell wall to external stimuli after CVR treatment (Figure 6A). The resistance of *A. alternata* mycelia to NaCl, CR, and SDS was weakened (Figure 6B). In response to H_2_O_2_, inhibition of the inside part of the mycelia was not significant but inhibition of the outside part of the mycelia was more significant after CVR treatment. This indicates that there were differences in the ability of the internal and external mycelia of *A. alternata* to respond to oxidative stress after CVR.

## 4. Discussion

Postharvest black mold rot infected by *A. alternata* is one of the most critical fungal decays encountered in goji berries during storage, resulting in tremendous economic losses (Wang et al., 2018, 2021). The defects of harmful to human health and environmental pollution in controlling Alternaria rot by using chemical fungicides promote the need to explore environmentally friendly acceptable sources of antifungal compounds. Essential oils are a promising alternative to synthetic fungicides for controlling postharvest fungal disease (Gonçalves et al., 2021). Previous studies have demonstrated that CVR can effectively inhibit the growth of *A. alternata in vitro* and control the black mold of goji berries after harvest (Abbaszadeh et al., 2014; Wang et al., 2019; Zhang et al., 2019). This study is to further explore the possible antifungal mechanism of CVR against the mycelial growth of *A. alternata*. Microscopy observations showed that CVR disrupted the cell wall of *A. alternata* by thinning, fracturing, and collapsing the mycelia (Supplementary Figure S7). This is consistent with the results of CVR treatment disrupting the cell wall of *Botryosphaeria dothidea* (Li J. et al., 2021; Li Q. et al., 2021). CWF can specifically bind chitin in the cell wall, which can be observed by fluorescence microscopy under blue fluorescence. After CVR treatment, the mycelial surface fluorescence was uneven, the mycelial interval was shortened, the mycelial septum was damaged, and the cell wall was obviously damaged. This showed that CVR can inhibit *A. alternata* by destroying the cell wall (Figure 1A). This phenomenon is consistent with the effect of cinnamaldehyde on *Geotrichum citri-aurantii* (a postharvest pathogen of citrus) reported by Ouyang et al. (2019). AKP is an enzyme that is produced in the cytoplasm and leaks into the periplasmic space. Generally, AKP releases from the fungal cells through the cell wall in which permeability is impaired (Yang et al., 2016). The cell wall permeability was significantly increased and the integrity of the cell wall of *A. alternata* was destroyed after CVR treatment (Figure 1B). CVR treatment of *A. alternata* resulted in the inability to maintain normal hypha morphology and affected normal growth of the hypha. FT-IR revealed that the -OH groups and protein-specific functional groups (amide I and amide II) in the mycelia of *A. alternata* were changed after CVR treatment (Figure 1C). Serrano et al. (2010) reported that the deflection of the -OH group was due to the substitution of some of the -H. In summary, the structural material on the surface of *A. alternata* was changed after CVR treatment, and this affected mycelial growth.

The fungal cell wall determines the complex shapes of fungi, and changes in cell shape underpin morphogenesis and cellular differentiation (Gow et al., 2017). Meanwhile, the cell wall is also the natural interface between the fungus and its environment, and plays a key role in the infection of hosts by fungi (Kou and Naqvi, 2016). Therefore, the integrity and construction of the cell wall are instrumental in the success of fungi. The cell wall has been extensively studied and is an antifungal target. Polysaccharides, which are the main component of the fungal cell wall, maintain the structure of the cell wall. Structural characterization of the cell wall showed that it comprised an outer layer of mannose and proteins, and an inner layer of β-glucan and chitin, close to the cytoplasmic membrane (Rappleye et al., 2007). In fungal cell walls, β-1,3-glucan and chitin are both indispensable predominant components; the cross-linked product of the two can improve the stability of the cell wall and protect the fungus from being effect during the infection process. Simultaneously, β-1,3-glucan and chitin were also found to be the main substances supporting the cell wall structure (Gow et al., 2017). Relative changes of polysaccharides in mycelia can be calculated by XPS (Dague et al., 2008). In this study, XPS showed that the content of C_Ps_ in the hyphal cell wall of *A. alternata* was significantly decreased after CVR treatment (Table 1). CVR reduced the content of polysaccharides in the cell wall and thus damaged the integrity of cell wall. The contents of chitin and β-1,3-glucan in *A. alternata* were decreased significantly after CVR treatment (Figures 2A,B). Meanwhile, CVR treatment reduced the activities of β-1,3-glucan synthase and chitin synthase (Figures 2C,D), while the activity of β-1,3-glucanase and chitinase was increased in *A. alternata* after CVR treatment (Figures 2E,F). Hopke et al. (2018) showed that fungal responses to stresses include altering their cell surfaces to enhance or limit immune recognition and responses, linking the two disparate fields of cell wall integrity and immunity. The fungal cell wall can escape host immunity by increasing the content of chitin, while the β-glucan content increases the strength of cell wall. CVR inhibited the production of β-1,3-glucan and chitin by inhibiting the activities of β-1,3-glucan synthase and chitin synthase and promoted the degradation of the two polysaccharides by increasing the activities of β-1,3-glucanase and chitinase. This result was consistent with the report of *Tulbaghia violacea* Harv. plant extract decreasing the chitin and β-1,3-glucan contents in *Aspergillus flavus* (Belewa et al., 2017). CVR treatment affected the integrity and stability of the cell wall of *A. alternata* by affecting the synthesis and metabolism of β-1,3-glucan and chitin in the cell wall. Comparison of the inside and outside parts of the mycelia revealed that CVR treatment had a more significant effect on the outer part of the mycelia. At sites of cell wall expansion, chitin synthases constitute a family of membrane-embedded enzymes that catalyze the synthesis of chitin, while β-1,3-glucan synthase catalyzes synthesis of β-1,3-glucan and transports it to the cell wall in the form of vesicles (Riquelme et al., 2018). Owing to different growth states, polysaccharide construction of the formed mycelia and at sites of cell wall expansion is different. Therefore, the effect of CVR treatment on the construction of chitin and β-1,3-glucan in the outside part of the mycelia was more intense.

To reveal the effect of CVR on polysaccharide metabolism and transportation, the outer mycelia treated with 0.06 μl/ml CVR were selected for transcriptomic analyses. The results showed the regulation of a large number of gene expression levels and metabolic processes by CVR-treated. Polysaccharides were the main substances constituting the cell wall, and the transcriptome data showed that CVR treatment had a significant effect on starch and sucrose metabolic pathways in *A. alternata*. There were eight DEGs involved in the decomposition of β-1,3-glucan and β-D-glucoside (Figures 5A,B). Glucan endo-1,3-β-D-glucosidase, glucan-1,3-β-glucosidase, and β-D-1,3-glucohydrolase can regulate the degradation of β-1,3-glucan (Donzelli et al., 2001; Belewa et al., 2017). GPI-anchored cell wall beta-1,3-endoglucanase EglC is the main gene that regulates the growth cycle of fungi and insufficient expression of this gene can cause defects in cell growth (Ramirez-Garcia et al., 2017). CVR treatment significantly affected the expression of regulatory genes related to glucan endo-1,3-β-D-glucosidase and glucan-1,3-β-glucosidase and significantly increased the expression of β-D-1,3-glucohydrolase regulatory genes, which accelerated the conversion of β-1,3-glucan and β-D-glucoside from polysaccharide to D-glucose (Henrissat and Bairoch, 1993). The upregulated expression of glucan endo-1,3-β-D-glucosidase and glucan-1,3-β-glucosidase was consistent with the increase in activity of β-1,3-glucanase. In addition, genes related to chitin anabolism were affected by CVR treatment. Chitin deacetylase-like protein can catalyze the hydrolysis of acetamido groups of *N*-acetylglucosamine units of chitin, producing glucosamine units and acetic acid (Mukherjee et al., 2019). The expression of the gene encoding this chitin deacetylase-like protein was increased in the CVR-treated samples, indicating that accelerated breakdown of chitin occurred in the hyphal cell wall after CVR treatment. The upregulated expression of this gene was also consistent with the increase in activity of chitinase. Chitin-binding protein can promote the synthesis of chitin, and the chitin-binding protein has an important role in the growth and development of infectious mycelia which has diverse functions in morphogenesis, pathogenesis, parasitism, nutrition, and immune regulation (Kuranda and Robbins, 1991). The upregulation of the gene encoding this enzyme indicates that CVR treatment affects the growth cycle of mycelia. The downregulated expression of the chitin synthase gene indicated that CVR treatment inhibited chitin synthesis. The gene expression data were consistent with the activity of chitin synthase. Therefore, CVR treatment affected the integrity of the cell wall of *A. alternata* by regulating the genes related to polysaccharide biosynthesis and metabolism of the cell wall.

Recent studies have begun to reveal the extraordinary influence of the fungal cell wall on many aspects of fungal physiology, particularly in the interaction of fungi with the environment and during the colonization of host tissues (Geoghegan et al., 2017). Mycelia treated with different concentrations of CVR showed growth inhibition in the media containing different stress substances. The results of these assays indicated that the function of the cell wall was weakened to resist perturbing stress, osmotic stress, and high-temperature stress after CVR treatment (Figure 6). This was congruent with the findings of Zhang et al. (2020). Concurrently, CVR treatment affected the infection function of *A. alternata* cell wall. Covalently linked cell wall protein was covalently cross-linked to the cell wall and was an important site for hyphal cell wall adhesion and recognition. The upregulation of this gene of covalently linked cell wall protein indicates that CVR treatment affects the infection and stress resistance of *A. alternata* mycelia. However, the inside part of the mycelia did not exhibit a significant inhibitory effect in the medium containing H_2_O_2_. The reason(s) underlying the differing response of different parts of the mycelia to oxidative stress requires further investigation.

## 5. Conclusion

In summary, CVR has a destructive effect on the cell wall of *A. alternata* by damaging and altering the integrity of the mycelial cell wall. Meanwhile, both the contents of cell wall polycarbohydrates containing chitin and β-1,3-glucan and the activities of the enzymes related to the biosynthesis of these polycarbohydrates were significantly decreased by CVR treatment, while the activities of chitinase and β-1,3-glucanase response of degrading the two polycarbohydrates were increased by CVR treatment. The transcriptomic analysis further revealed that CVR influenced the polycarbohydrate metabolism pathways, especially starch and sucrose metabolism signaling pathways. In addition, CVR treatment inhibited the stress resistance of *A. alternata in vitro*. These findings implicated that CVR might exhibit its antifungal activity against *A. alternata* by interfering with the construction of the cell wall and therefore lead to the destruction of cell wall integrity and function. However, the inhibition mechanism of CVR treatment on the cell wall of *A. alternata* needs to be further studied by combining proteomics or metabolomics.

## Data availability statement

The datasets presented in this study can be found in online repositories. The names of the repository/repositories and accession number(s) can be found below: BioProject: accession number: PRJNA923470.

## Author contributions

LZ, JW, and HZ contributed in conceptualization, methodology, formal analysis, investigation, writing original draft, writing review and editing, and supervision. JW contributed in validation, resources, funding acquisition, and experimental materials. PW, CW, YZ, LH, and RW contributed in investigation. All authors contributed to the article and approved the submitted version.

## Funding

This work was supported by Natural Science Foundation of Ningxia Province (2020AAC03255) and Natural Science Foundation of China (32060565).

## Conflict of interest

The authors declare that the research was conducted in the absence of any commercial or financial relationships that could be construed as a potential conflict of interest.

## Publisher’s note

All claims expressed in this article are solely those of the authors and do not necessarily represent those of their affiliated organizations, or those of the publisher, the editors and the reviewers. Any product that may be evaluated in this article, or claim that may be made by its manufacturer, is not guaranteed or endorsed by the publisher.
