# Supplementary material for: Inhibitory effect of carvacrol against Alternaria alternata causing goji fruit rot by disrupting the integrity and composition of cell wall
Source: Front Microbiol. 2023 Feb 20;14:1139749. doi: 10.3389/fmicb.2023.1139749 (PMC9986456; doi:10.3389/fmicb.2023.1139749)
Supplement: Supplementary file 1 [file Data_Sheet_1.docx]

Supplementary Material

# Supplementary Figures and Tables

**Supplementary**
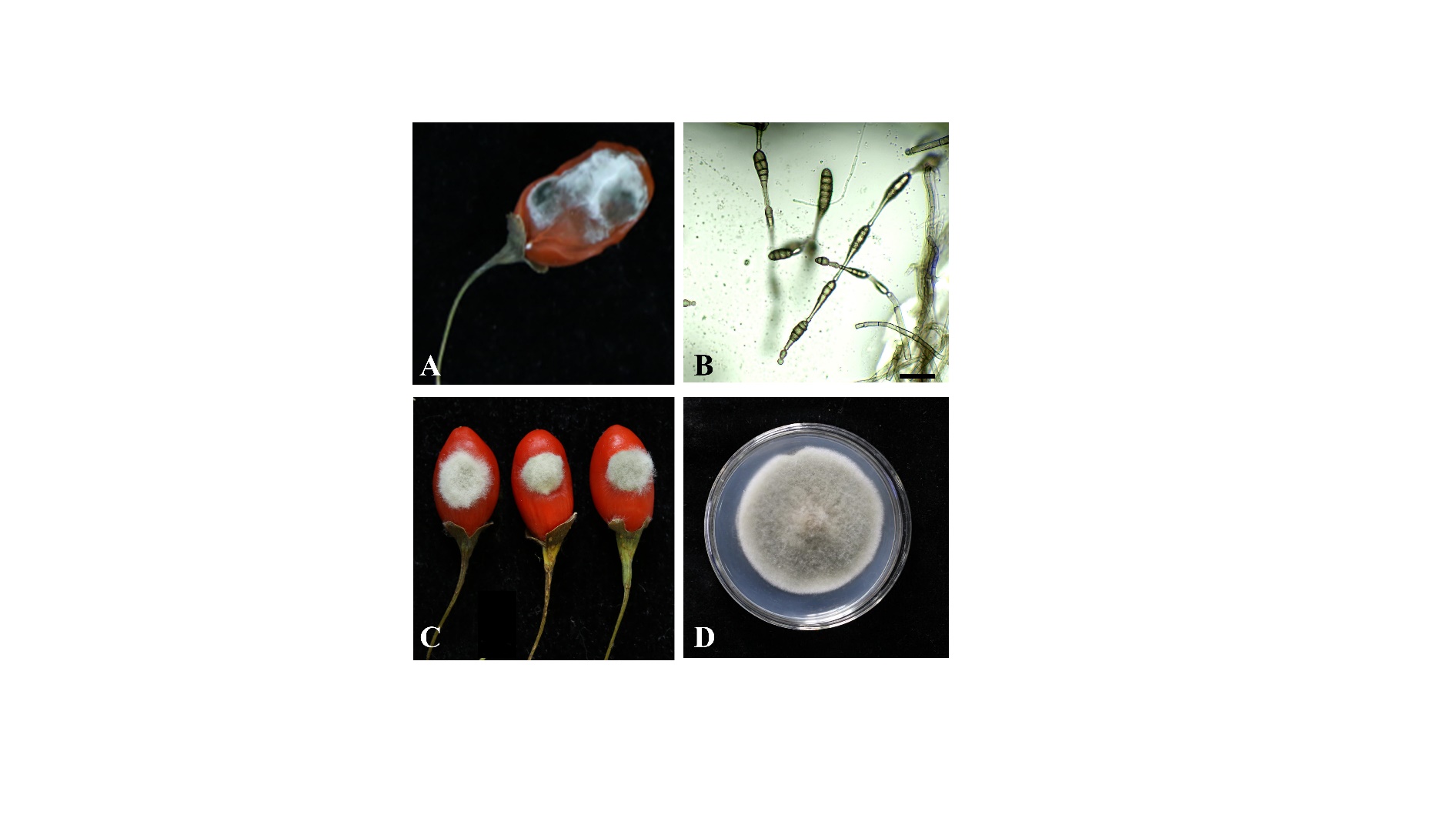
**Figure S1.** Signs and morphology of the fungal pathogen in Goji berry. (A) Symptoms of the disease; (B) Conidiophore; (C) Stab inoculation; (D) Mycelium colonies cultured on PDA plate.

During the investigation, light gray to dark brown mycelium was observed on the surface of the berries, and it rapidly spread to the whole fruit (Figure S1A). This fruit rot was typically observed after storage for 4 to 6 days after harvest at room temperature (about 25°C). Single or short-chain conidia were observed: they were multicellular, beaked or beakless, obclavate to obpyriform, brown, 14.11–43.07 μm (average, 24.95 μm) in length, and 6.27–16.33 μm (average, 9.72 μm) in width, with 0–3 longitudinal and 2–6 transverse septa (n = 50) (Figure S1B). Moreover, according to the results of Koch’s postulates experiment, the disease symptom on inoculated fruits with the isolate was identical to that of the nature symptom (Figure S1C). On PDA medium, the colonies initially appeared white later turned dark brown to black, with a white margin and abundant aerial hyphae (Figure S1D). These morphological features matched those of *Alternaria alternata* (Fr.) Keissl ([Simmons, 2007](https://apsjournals.apsnet.org/doi/10.1094/PDIS-09-17-1430-PDN#b2)).

Simmons, E. G. (2007). CBS Fungal Biodiversity Centre. Utrecht, The Netherlands.

**Supplementary Figure 2.** Neighbor-joining phylogenetic tree based on *rDNA-ITS* gene sequences.

**MN653245.1 LTS7-1802**

KC692221.1 *Alternaria alternata* ML356

MK757897.1 *Alternaria alternata* J7

KT192394.1 *Alternaria alternata* ZG-2-3-4

EU520199.1 *Alternaria bokurai* NW549

EU732734.1 *Alternaria tenuis* NW678C

HQ680452.1 *Penicillium oxalicum* Po-5

MN249893.1 *Penicillium oxalicum* 8A8

GU982311.1 *Gibberella moniliformis* PUMCH10XB00173

EU151467.1 *Gibberella moniliformis* A1

KY075855.1 *Fusarium proliferatum* MEMO556677

FN868470.1 *Fusarium proliferatum* BLE1

100

98

73

99

100

57

81

99

75

0.05

The 548-bp long sequence of the *ITS* region was found to have 100% homology with reported sequences of *A. alternata* isolates with accession numbers KC692221.1, MK757897.1, and KT192394.1.

**Supplementary Figure 3.** Neighbor-joining phylogenetic tree based on *tub2* gene sequences

**MN702782.1 LTS7-1802**

KY609181.1 *Alternaria alternata* ALT-1

MK558217.1 *Alternaria alternata* 61/4

MF070269.1 Alternaria alternata BAS 110

MF070256.1 *Alternaria tenuissima* CBS124278

MF070250.1 *Alternaria tenuissima* CBS124283

KJ883438.1 *Alternaria japonica* CBS 118390

HQ413317.1 *Alternaria* *solani*

MF070254.1 *Alternaria gaisen* CBS118488

MF070247.1 *Alternaria radicina* CBS112003

Y17082.1 *Alternaria lini* CBS 106.34

JQ314398.1 *Pyrenophora teres* f.

53

98

100

73

53

59

13

16

20

0.02

The 1048-bp *tub2* sequence showed 100% homology with *A. alternata* sequences with accession numbers KY609181.1, MK558217.1, and MF070269.1.

**Supplementary Figure 4.** Neighbor-joining phylogenetic tree based on *endoPG* gene sequences.

**MN698284.1 LTS7-1802**

KY963795.1 *Alternaria alternata* LH-4

MH003581.1 *Alternaria alternata* FPH2015597

MF964627.1 *Alternaria alternata* NAS-AA5

KP789521.1 *Alternaria alternata* P117

KP789520.1 *Alternaria alternata* CY17

AY295026.1 *Alternaria citriarbusti* SH-MIL

KP789510.1 *Alternaria tenuissima* P60

KY969575.1 *Alternaria tenuissima* W26H

AY295028.1 *Alternaria arborescens* EGS 39-128

EF504123.1 *Alternaria arborescens* PIST0462

XM 028648000.1 *Alternaria arborescens* AA0111

61

78

85

47

96

58

42

62

44

0.001

The 489-bp sequence of *endoPG* displayed 100% homology with the *A. alternata* isolate with accession number KY963795.1 deposited in GenBank.

**Supplementary Figure 5.** Neighbor-joining phylogenetic tree based on *Altal* gene sequences.

JQ646408.1 *Alternaria resedae* CBS

KY700705.1 *Alternaria resedae* UEM3913

MF958692.1 *Alternaria citri* SXWMR4

MF958691.1 *Alternaria citri* SXWMR3

MF581211.1 *Alternaria viniferae* JZB311086

MF581210.1 *Alternaria viniferae* JZB311081

MH567108.1 *Alternaria alternata* HMCH-9

MG250644.1 *Alternaria alternata* RG3

KX894537.1 *Alternaria alternata* SRLS-1

**MN702781.1 LTS7-1802**

MF381753.1 *Alternaria alternata* PPRI:13491

NW 020171353.1 *Trichoderma gamsii* T6085

89

43

61

55

76

93

0.1

The 503-bp sequence of the *Alta1* gene showed 100% homology with *A. alternata* isolates with accession numbers MH567108.1, MG250644.1, KX894537.1, and MF381753.1.


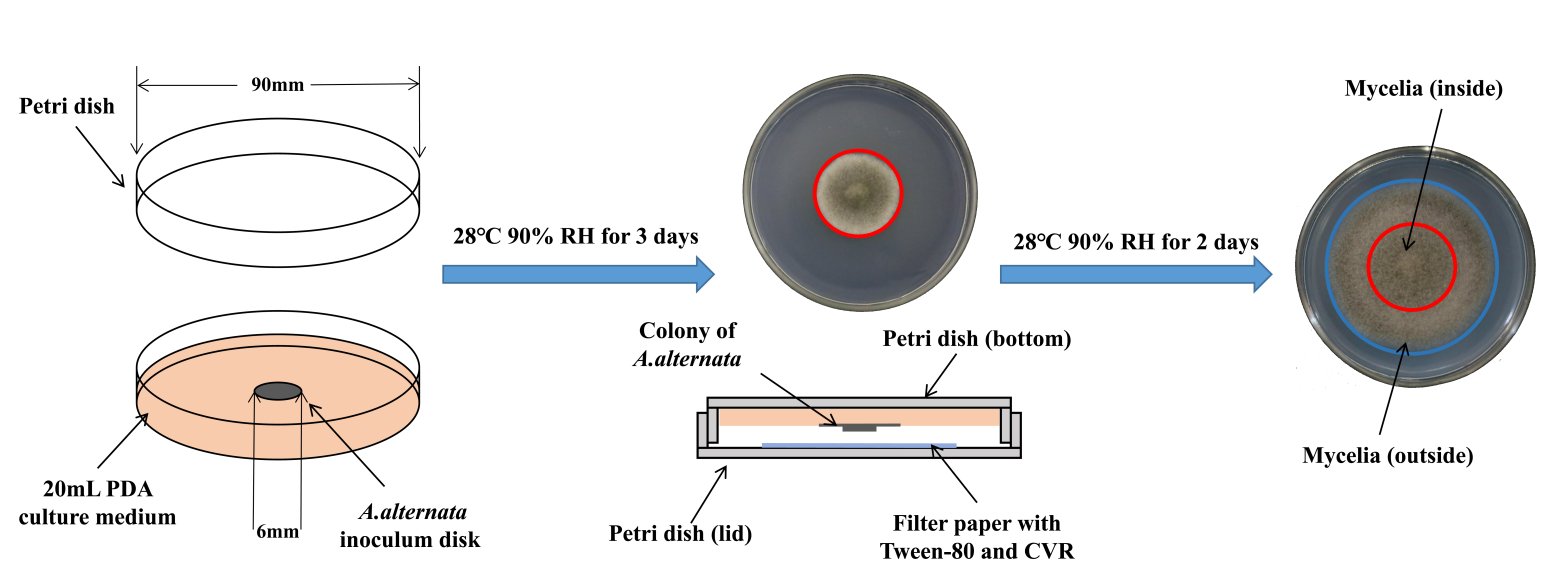


## Supplementary Figure 6. Experimental design for preparation of CVR treatments of *A. alternata*


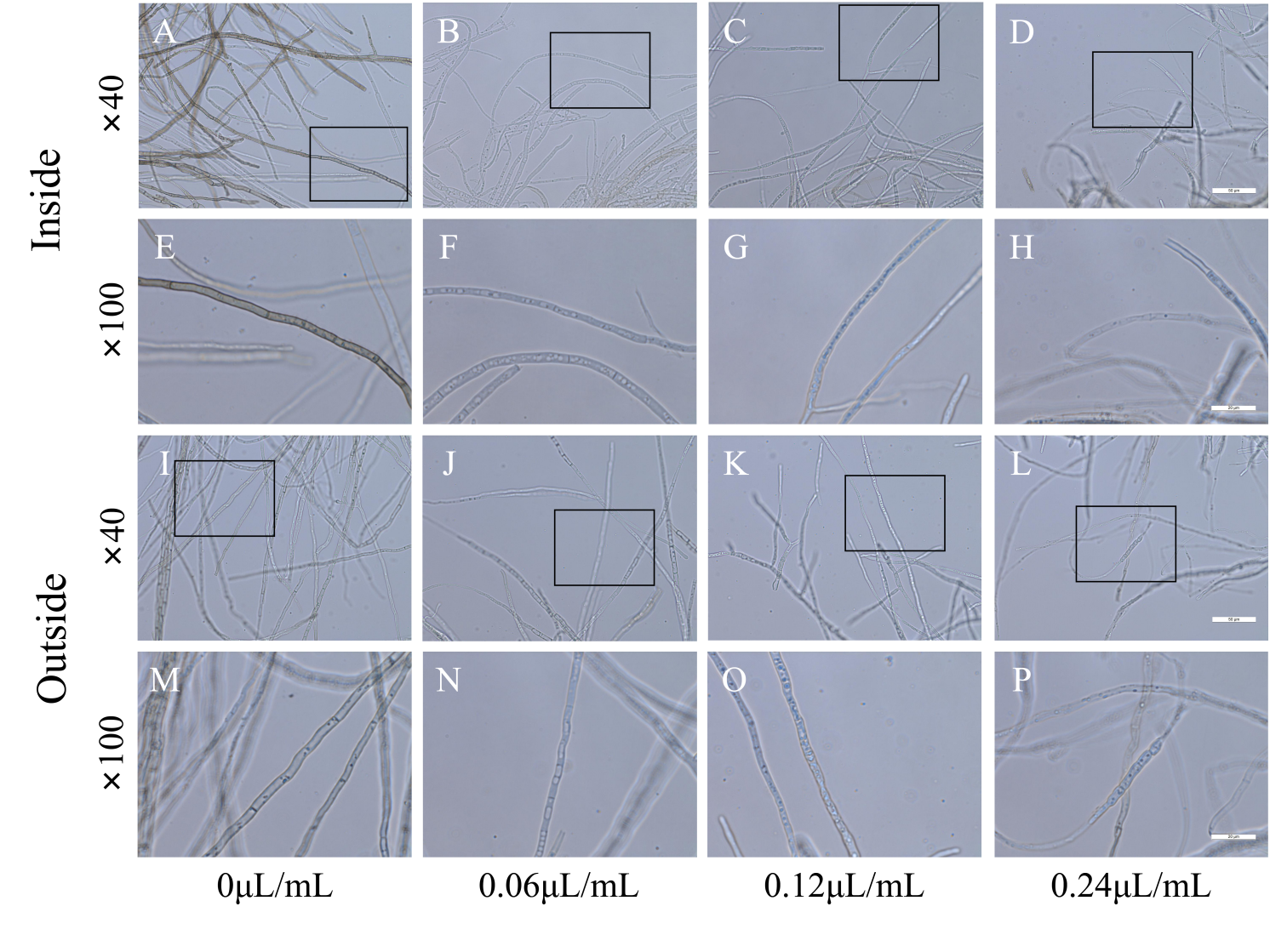


## Supplementary Figure 7. Microscopy observation of *A. alternata.* CK treatment of inside (A and E) and outside (I and M) parts of mycelia; 0.06 μL/mL CVR treatment of inside (B and F) and outside (J and K) parts of mycelia; 0.12 μL/mL CVR treatment of inside (C and G) and outside (K and O) parts of mycelia; 0.24 μL/mL CVR treatment of inside (D and H) and outside (L and P) parts of mycelia.


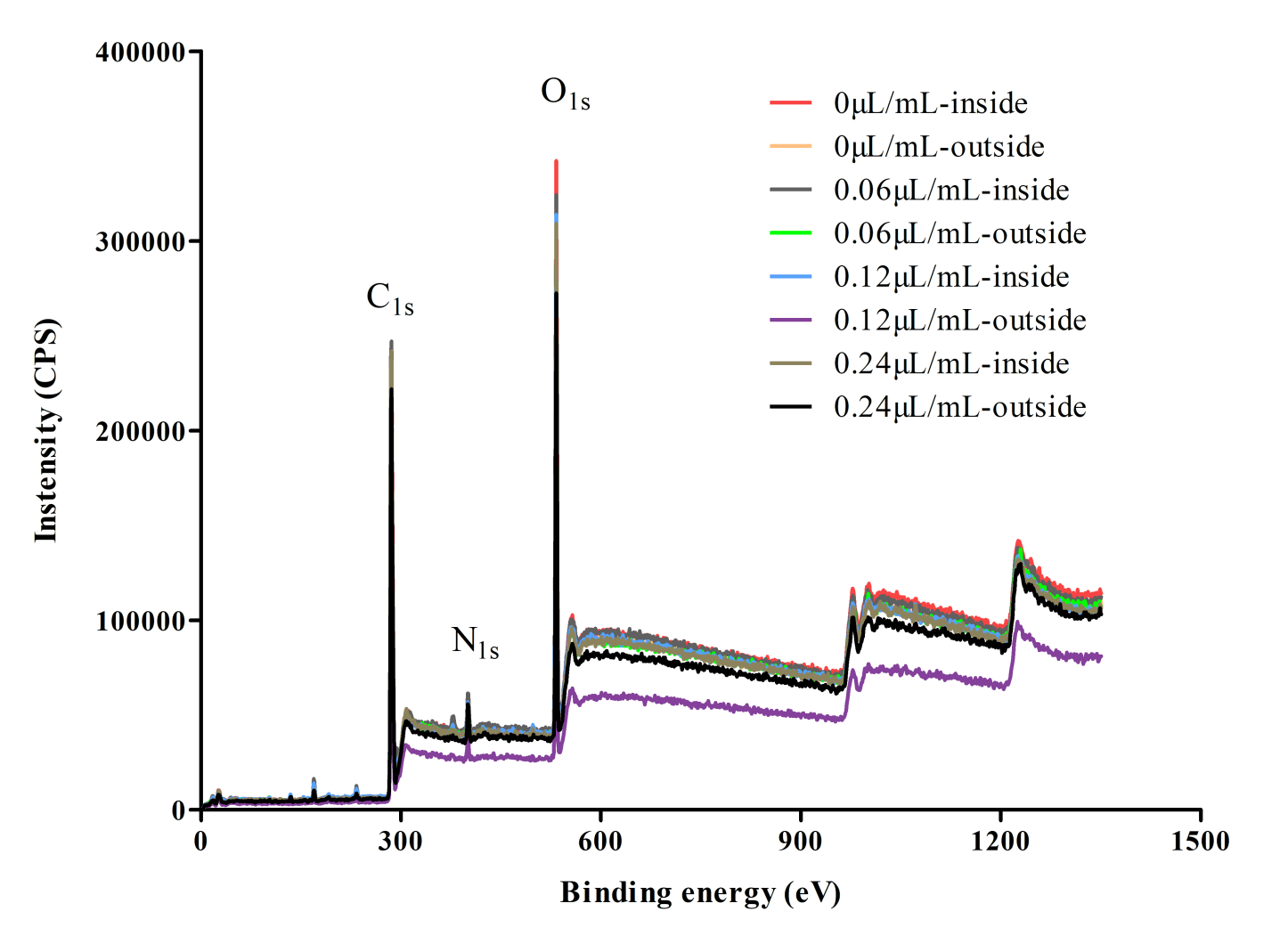


**Supplementary Figure 8.** X-ray photoelectron spectroscopy (XPS) of *A. alternata*

**Supplementary Table 1.** Real-time PCR primer sequences for differentially expressed genes

| Gene | Primers of positive-sense and anti-sense (5‟-3‟) | |
| --- | --- | --- |
| β-tubulin | F-TCATACTTCGTTGAGTGGAT | R-CTGGATGGAGGTGGAGTT |
| CC77DRAFT_940462 | F-CTTCTTTGTTACTGGTTCGCT | R-CTGGGATGTTAGGTCGTTG |
| CC77DRAFT_1061482 | F-CGGCATCGTTCTTATTCGAT | R-GAAGACGCACTTCGTGTCAA |
| CC77DRAFT_926154 | F-TTGAGGACGGAAAGTTGACC | R-CCTGGATATGGATGGGAATG |
| CC77DRAFT_598231 | F-TTACTGGGACGAGGTTGG | R-GGTGTTGTTCTCCTTCGG |
| CC77DRAFT_971887 | F-AACAGCGTTGAGGAGGAAGA | R-GCAGTCTTTCCGGTGACAAT |
| CC77DRAFT_411420 | F-GCACCTTCGAGTACCGAGAC | R-GCCTGTACCGGTCCAATAGA |
| CC77DRAFT_1083497 | F-CTGGCTATTGGGACAGCATT | R-GTCGACGTCCCTTTGTTTGT |
| CC77DRAFT_190672 | F-CACCTTCAAGTGGGACACCT | R-GCGCAAGGTTGGGAATAGTA |
| CC77DRAFT_1019398 | F-GGAGTATGGCGAACCACTGT | R-CGAGTCATTCCACCGACTTT |
| CC77DRAFT_1057870 | F-CCGACTGCTTGATTGACTT | R-TAGGTGATGGCGACTGTTC |
| CC77DRAFT_983002 | F-CTCCTGATGAATGGACTCTC | R-CGATAGTGGTGGTGTTGATA |
| CC77DRAFT_1082319 | F-GGTTCAAGGGCAACATGACT | R-ACTCGCCAACAGTGACTCCT |
| CC77DRAFT_237249 | F-CAGCAGCAACAACACCAA | R-AAGACGACAGACAAAGCCA |
| CC77DRAFT_1018725 | F-TCCGCTATTGGAGAGCCTA | R-TCGTAACTGTGAGGCGTTTG |
